# Evaluation of cell count and classification capabilities in body fluids using a fully automated Sysmex XN equipped with high-sensitive Analysis (hsA) mode and DI-60 hematology analyzer system

**DOI:** 10.1371/journal.pone.0195923

**Published:** 2018-04-26

**Authors:** Hiroyuki Takemura, Tomohiko Ai, Konobu Kimura, Kaori Nagasaka, Toshihiro Takahashi, Koji Tsuchiya, Haeun Yang, Aya Konishi, Kinya Uchihashi, Takashi Horii, Yoko Tabe, Akimichi Ohsaka

**Affiliations:** 1 Department of Clinical Laboratory, Juntendo University Hospital, Tokyo, Japan; 2 Department of Clinical Laboratory Medicine, Juntendo University School of Medicine, Tokyo, Japan; 3 Department of Next Generation of Hematology Laboratory Medicine, Juntendo University Graduate School of Medicine, Tokyo, Japan; 4 Sysmex, Hematology-Product Engineering, Product Development, Kobe, Japan; 5 Department of Transfusion Medicine and Stem Cell Regulation, Juntendo University Graduate School of Medicine, Tokyo, Japan; The Ohio State University, UNITED STATES

## Abstract

The XN series automated hematology analyzer has been equipped with a body fluid (BF) mode to count and differentiate leukocytes in BF samples including cerebrospinal fluid (CSF). However, its diagnostic accuracy is not reliable for CSF samples with low cell concentration at the border between normal and pathologic level. To overcome this limitation, a new flow cytometry-based technology, termed “high sensitive analysis (hsA) mode,” has been developed. In addition, the XN series analyzer has been equipped with the automated digital cell imaging analyzer DI-60 to classify cell morphology including normal leukocytes differential and abnormal malignant cells detection. Using various BF samples, we evaluated the performance of the XN-hsA mode and DI-60 compared to manual microscopic examination. The reproducibility of the XN-hsA mode showed good results in samples with low cell densities (coefficient of variation; % CV: 7.8% for 6 cells/μL). The linearity of the XN-hsA mode was established up to 938 cells/μL. The cell number obtained using the XN-hsA mode correlated highly with the corresponding microscopic examination. Good correlation was also observed between the DI-60 analyses and manual microscopic classification for all leukocyte types, except monocytes. In conclusion, the combined use of cell counting with the XN-hsA mode and automated morphological analyses using the DI-60 mode is potentially useful for the automated analysis of BF cells.

## Introduction

Cell density counting and cell type differentiation in body fluid (BF) samples are essential for determining therapeutic approaches to various diseases. For example, analysis of cell density and leukocyte type in cerebrospinal fluid (CSF) is essential for the treatment of meningitis and encephalitis [1]. Detection of malignant cells in ascites and pleural effusion is essential for disease staging. Although BF examinations must be conducted as soon as possible, manual microscopic examination and cell counting is time consuming, and the diagnosis can be affected by the individual examiner’s skill level [2]. Therefore, new analysis systems have recently been developed to automatically count cells using hematological analyzers [3–5]. However, developing new algorithms is still technically challenging. For example, cell counting in samples with extremely low cell concentration yields high imprecision. Differentiation of malignant cells also requires sophisticated algorithms due to variability in cell morphology, size, and intracellular content. To overcome these technological difficulties, high-sensitive analysis mode (hsA mode, XN-hsA) and the automated digital cell imaging analyzer DI-60 have been developed and incorporated into the automated hematology analyzer XN series (XN; Sysmex, Kobe, Japan) [6]. Leukocyte morphology, which is useful to determinate between bacterial and viral infections based on the relative proportions of neutrophils and lymphocytes as well as to detect abnormal malignant cells, can be semi-automatically analyzed with DI-60 on cytospin slides stained using the May-Grünwald Giemsa method [7]. This study investigated the feasibility and accuracy of the XN-hsA mode and DI-60 using various BF samples.

## Materials and methods

### Sample collection

This study was conducted using patient samples, and approval was obtained from the Juntendo University Hospital Medical Ethics Committee. Written informed consent was waved by the ethical committee since all samples were de-identified for analyses. The original data are available upon request (http://www.juntendo.ac.jp/english/research.html). The samples included 60 CSF samples and 60 other BF samples (51 pleural effusions, 8 ascites, 1 pericardial effusion) that were sent to the Clinical Laboratory department, Juntendo University Hospital (Tokyo, Japan). Cytospin slides used for the cell classification were prepared using a Shandon Cytospin 4 Cytocentrifuge (Thermo Fisher Scientific, Waltham, MA) and then were stained with May-Grünwald Giemsa using HEG-NST (Sysmex). For the control method, manual cell counting using the Fuchs-Rosenthal method was performed in accordance with the Clinical and Laboratory Standards Institute (CLSI) H56-A guidelines using 200 cells on the same slide that was used in the DI-60 analysis.

### Within-run precision

The XN-hsA mode has several new features to increase its accuracy: (1) providing a 4-part differential count; (2) utilizing flow cytometry technique for counting RBCs; (3) aspirating more sample volume (180 μL) than the XN-BF mode; and (4) counting twice as many cells [8]. To evaluate within-run precision, cell counts were repeated five times for each primary BF sample using different cell densities. The mean cell density and coefficient of variation (% CV) were calculated and compared between the XN-hsA mode and the XN-BF mode.

### Dilution linearity

To evaluate the overall linearity of the hsA mode, CSF samples were sequentially diluted with phosphate-buffered saline (PBS). The diluted samples were analyzed using the XN-hsA and XN-BF modes. The measured cell numbers were plotted against the theoretical values, and the plots were fitted with a linear regression method.

### Correlation between the manual microscopic method and DI-60

To evaluate the feasibility of cell classification utilized an automated digital cell imaging analyzer DI-60, we analyzed 200 leukocytes in various body fluid samples. Each slide was pre-classified by the DI-60 and subsequently post-classified by an experienced medical technologist (H.T.) who was proficient in hematopathology and cytopathology. A senior clinical pathologist (Y.T.) supervised the results. Collection and analysis of the BF samples which were performed in compliance with CLSI guideline H56-A (CLSI, Body Fluid Analysis for Cellular Composition; Approved Guideline [CLSI Document H56-A], Wayne: CLSI; 2006). The post-classification results using DI-60 in CSF (34 samples) and other BF samples (60 samples) were compared to the results of manual microscopic method (200 cell counts) performed by H.T. Correlation studies were conducted for neutrophils, lymphocytes, eosinophils, and monocytes.

### Statistical processing

The correlation between the two different methods was evaluated using the Pearson product-moment correlation coefficient. Statistical analyses were performed with Microsoft Excel 2007 (Microsoft, Redmond, WA).

## Results

### Within-run precision

**Fig 1:**
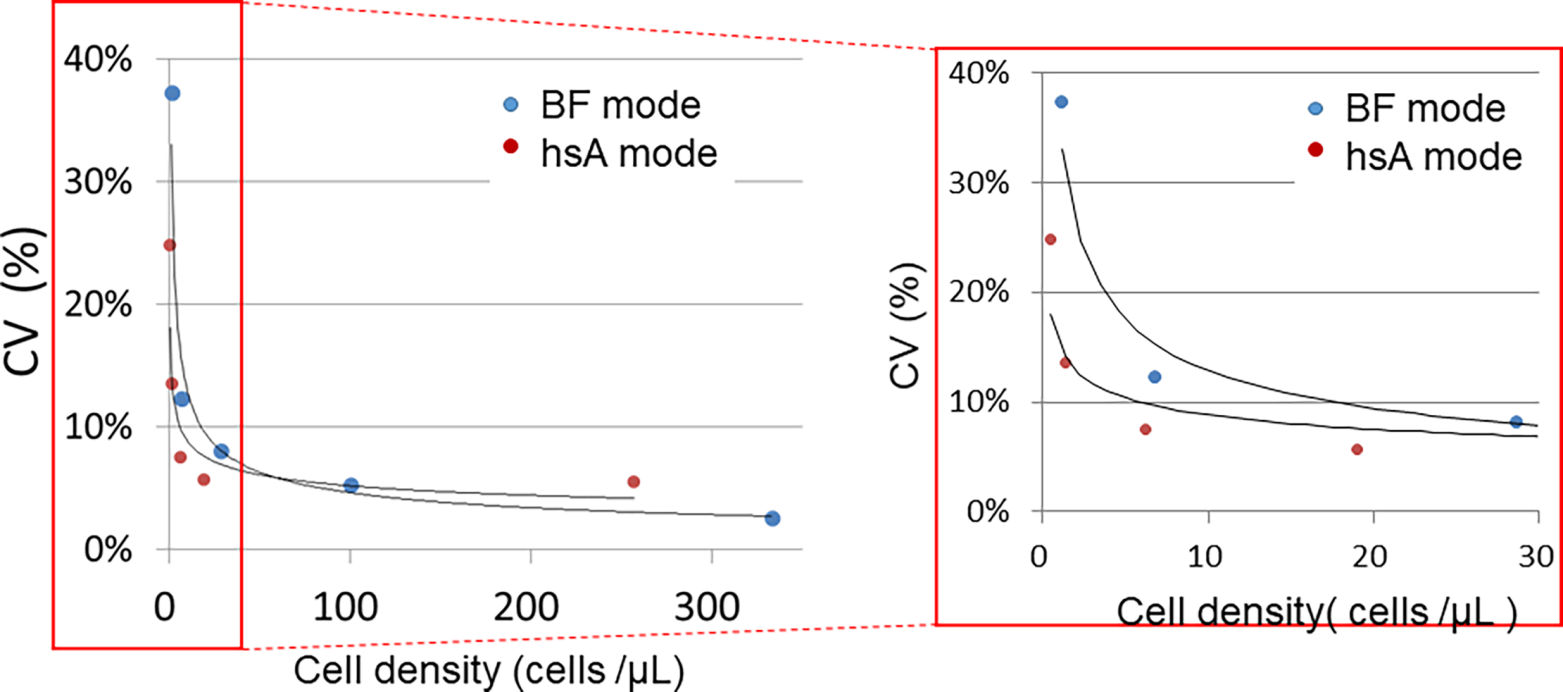
Coefficient of variations (% CV) measured with the XN-hsA and XN-BF modes as a function of CSF sample cell density. The right panel shows the magnified image of the portion of the plot (in the red rectangle).

CSF samples with various cell densities were measured five consecutive times using the XN-hsA and XN-BF modes. **Table 1** shows the mean cell densities and CVs. In both modes, the CVs were inversely correlated with cell density. To compare the precision of these two methods, the CVs were plotted against the cell densities (**Fig 1**). In the low cell density range, the CVs using the XN-hsA mode were smaller than for XN-BF (7.5% vs. 12.3% at the cell density of 6 cells/μL).

**Table 1:** Within-run precision. The mean cell number and coefficient of variations (% CV) of ten different CSF samples were calculated five consecutive times with the XN-hsA and the XN-BF modes.

XN-hsA mode			XN-BF mode		
Sample#	Mean (cells/μl)	CV (%)	Sample#	Mean (cells/μl)	CV (%)
1	256.7	5.6	6	333	2.6
2	19.0	5.7	7	100.2	5.3
3	6.2	7.5	8	28.6	8.0
4	1.3	13.6	9	6.8	12.3
5	0.5	24.8	10	1.2	37.3

### Dilution linearity

**Fig 2:**
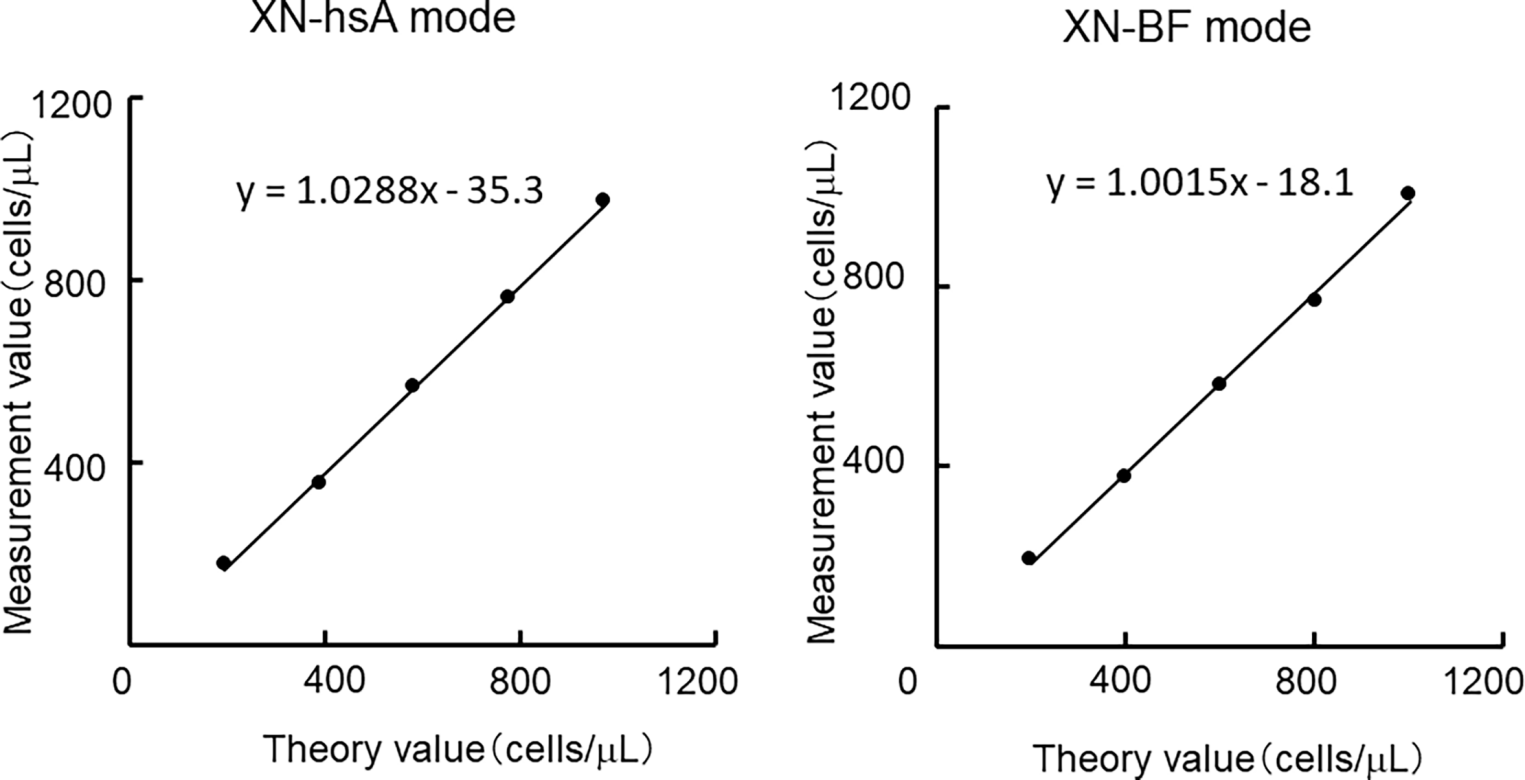
Plots of cell counts against the theoretical cell densities using serially diluted body fluid samples measured with the XN-hsA and XN-BF modes.

To validate the linearity of the XN-hsA mode, cell densities were measured using sequentially diluted samples in the hsA and BF modes. **Fig 2** shows the plots of the measured values against the theoretical values. The plots were fitted using linear regression. Compared to the theoretical values, good linearity was obtained for up to 938 cells/μL in the XN-hsA mode (r = 0.999) and up to 1,104 cells/μL in the XN-BF mode (r = 0.999).

### Accuracy of the XN-hsA and the XN-BF modes compared to the manual microscopic examinations

**Fig 3:**
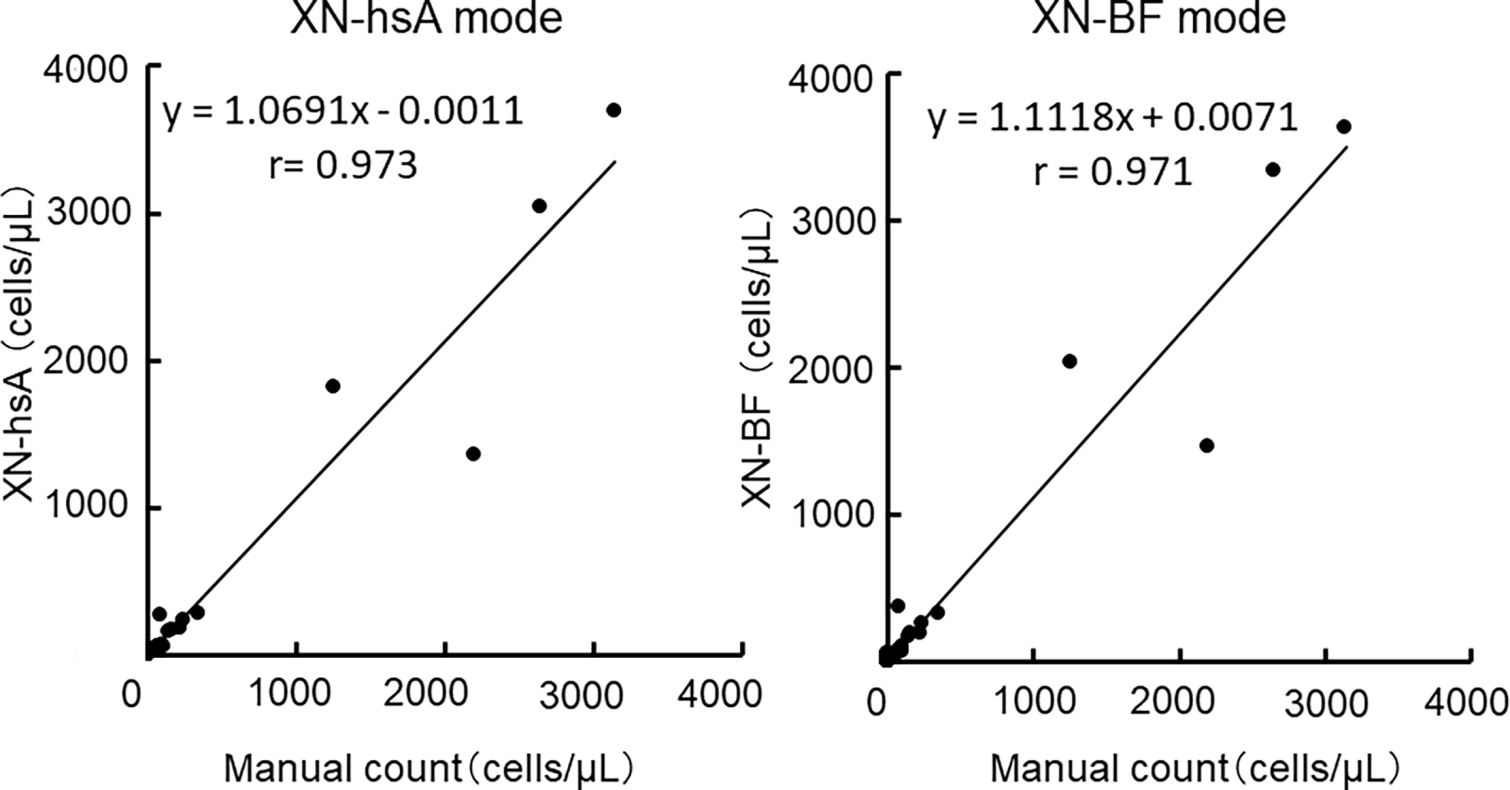
Correlation of cell density analyses between the XN-hsA and XN-BF modes and manual microscopic examination using CSF samples (n = 60). The raw data are shown in S1 Table.

Next, the accuracy of these two methods was compared to manual microscopic examination. **Fig 3** shows the plots of leukocyte counts in CSF samples (n = 60) measured with the two automated methods, XN-hsA and XN-BF modes, against the manual microscopic examination. Each plot was fitted with a linear regression, and both modes showed good agreement with the manual microscopic method. The raw data are shown in S1 Table.

### Correlation between the digital cell imaging DI-60 and the manual microscopic examinations

**Fig 4:**
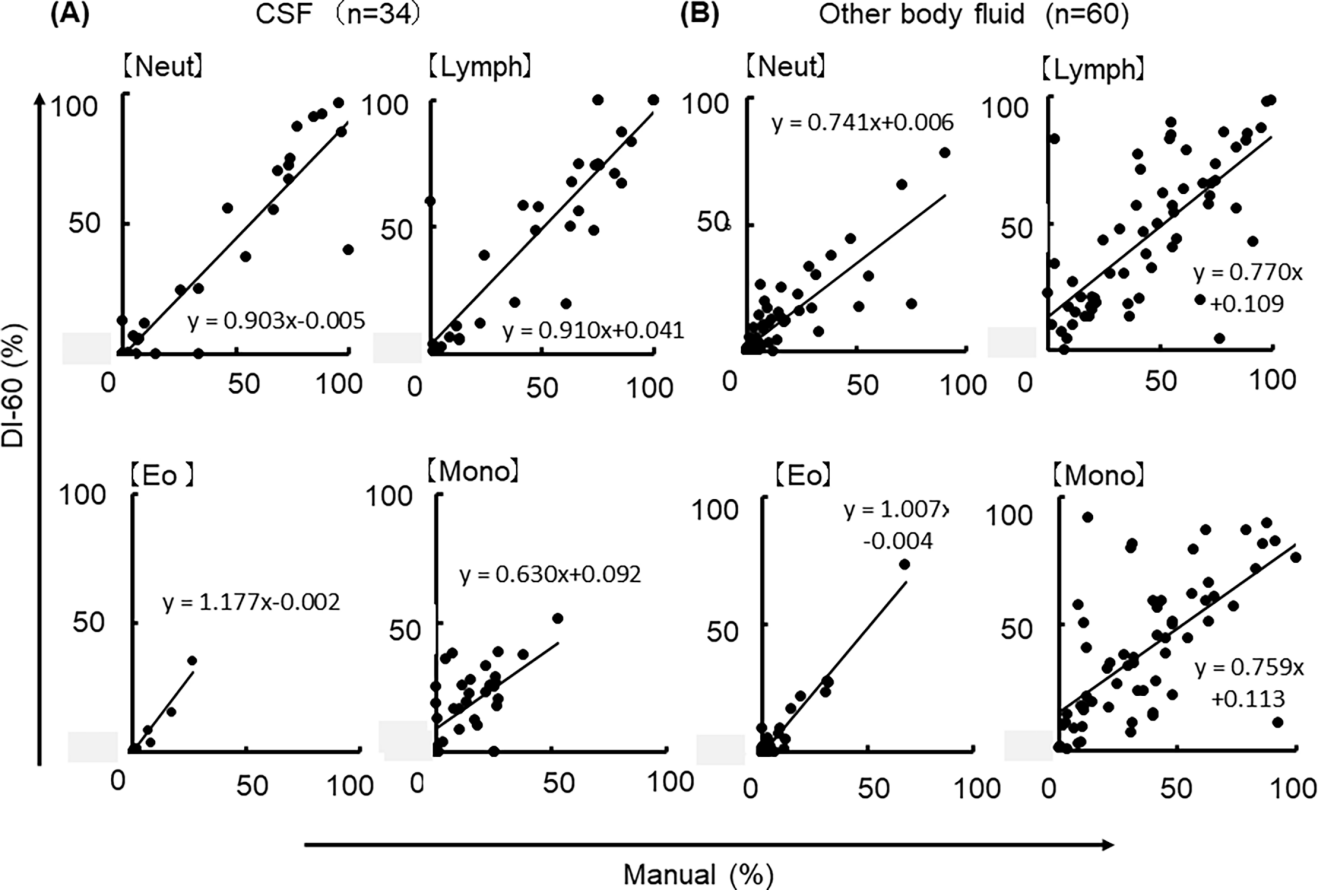
(A) Comparison of the classifications of four different leukocyte types using DI-60 and manual microscopic examination in CSF samples (n = 34). Neut depicts neutrophils; Lymph, lymphocytes; Eo, eosinophils; Mo, monocytes. (B) Classifications of four different leukocyte types using the DI-60 and manual microscopic examination in other body fluid samples (n = 60). The raw data are shown in S2 Table.

Finally, we investigated the feasibility of the digital cell imaging analyzer DI-60 by comparing with microscopic examinations using CSF (n = 34) and other BF samples (n = 60). The cells were classified into four different cell types (neutrophil, lymphocyte, eosinophil and monocyte). **Fig 4A** shows the correlation between morphological cell classification through DI-60 (post-classification) and manual microscopic examination in CSF samples. Each plot was fitted with a linear regression analyses, which yielded the following coefficients: r = 0.743 for neutrophils; r = 0.819 for lymphocytes; r = 0.770 for eosinophils; and r = 0.778 for monocytes. In the other body fluid samples, including ascites and pleural effusion, the correlation between the DI-60 (post-classification) and manual microscopic examinations were r = 0.973 for neutrophils; r = 0.939 for lymphocytes; r = 0.803 for eosinophils; and r = 0.638, for monocytes (**Fig 4B**). In both samples, good correlations were found between the two methods with the exception of monocytes. In non-CSF BF samples, the proportion of neutrophils and lymphocytes obtained using DI-60 tended to be lower than with the manual microscopic method. The raw data are shown in S2 Table.

## Discussion

Many researchers and manufacturers have attempted to develop new automated analyzing systems to measure cells in BF to replace manual examination which are time- and labor-consuming [9], and several new analysis algorithms have been invented that can be incorporated with automated hematology analyzers [6, 10, 11]. In this study, we evaluated the feasibility and accuracy of the most recently developed BF analyzing mode, XN-hsA, and morphology classifying imaging system, DI-60, using primary BF samples and comparing the results with manual microscopic examination. To the best of our knowledge, this is the first study that determined the within-run precision of XN-hsA in primary CSF samples with low leukocyte concentrations.

### Feasibility and accuracy of the XN-hsA

Cell counting in various BF samples by conventional automated hematology analyzers has been challenging, particularly when the cell density is extremely low. For example, the diagnostic threshold of leukocyte counts for meningitis in CSF samples is only 5 cells/μL, and there is large variability of cell counts in this range [1]. The cell density where the CV exceed 20% is arbitrarily defined as the lower limit of detection (LoQ), and this has been used to compare analyzers [3]. In our previous study using Sysmex XE-5000, the predecessor of the XN series, the CV in the measurement of CSF with a leukocyte density of 7.6 cells/μL was 27.9% [10]. By contrast, the CVs for similar samples with XN-hsA was less than 10%. This agreed with the detection limits of leukocyte densities reported by Fleming *et al*. [6]. These results indicate that XN-hsA mode can result in greater accuracy for BF laboratory examinations compared to the previous version. One issue is that the XN-hsA mode requires a larger sample volume (200 μL) than the XN-BF mode (88 μL). Although this can be problematic if the sample volume is limited, as it can be with CSF, further technical improvement is warranted.

### Feasibility and accuracy of the DI-60

In our study, the results of cell classification in BF samples using DI-60 showed good correlation with manual microscopic examinations for all of the cells except monocytes. This is likely due to the morphological complexity of monocytes; the morphology of monocytes is sometimes similar to macrophages since these cells share the same lineage and their morphological changes are continuous. Furthermore, the preparation of cytospin slides has several methodologic limitations [12]. For example, the morphologically degenerated cells during the centrifugation procedure to prepare cytospin slides were classified as smudge cells by the DI-60 system, which is another possibility that cause the relatively low correlation in with manual microscopic examination. Poor detection rate of monocytes in body fluid indicates the limitation of morphological examinations. Currently, monocytes can be differentiated using flowcytometry using CD markers. However, this is an expensive method and lots of labor are required. To overcome this issue, development of algorithms for more detailed differentiation of mononuclear cells in body fluid by the automated hematology analyzer might offer a possible solution.

Although the detection of malignant cells, which is clinically important to determine the appropriate course of treatment, is beyond the scope of this study, we are planning the further studies with larger sample sizes including malignant BF samples. In addition, we intend to evaluate the clinical efficacy of the novel combining flow-through cellular analysis using the XN-hsA mode with a new gating algorithm and DI-60 digital image analysis for the detection of malignant cells.

There were several limitations to this study. This was a single center study, the number of samples is relatively small, and the samples did not include any malignancies. We evaluated the accuracy of XN-hsA and DI-60 by comparing the manual microscopic examination, which is considered the gold standard. However, manual microscopic examination itself has several limitations including inter- and intra-examiner variance, inconsistent staining, and cell deformities due to the sampling procedure. For example, whereas the LoQ we detected for the hsA mode was approximately 5 cells/μL, the LoQ of the manual microscopic leukocyte counts has been reported to be 10 cells/μL [3] which is even higher than the one of hsA. Therefore, the comparison among these different methods can be affected by many unavoidable factors.

Even considering these limitations, we conclude that the combined use of XN-hsA mode and DI-60 equipped in an automated hematology analyzer can potentially provide physicians with prompt and accurate diagnostic information for the treatment of various diseases. However, further technological improvement is needed to improve the accuracy of dysmorphic cell detection.

## Supporting information

S1 Table The raw data for Fig 3. (DOCX)

S2 Table The raw data for Fig 4. (DOCX)
