# Supplementary material for: Evaluation of cell count and classification capabilities in body fluids using a fully automated Sysmex XN equipped with high-sensitive Analysis (hsA) mode and DI-60 hematology analyzer system
Source: PLoS One. 2018 Apr 26;13(4):e0195923. doi: 10.1371/journal.pone.0195923 (PMC5919509; doi:10.1371/journal.pone.0195923)
Supplement: S1 Table — (DOCX) [file pone.0195923.s001.docx]

**S1 Table. Raw data for Fig 3**

| Sample # | manual count | XN (hsA) | XN (BF) | (cells/uL) |
| --- | --- | --- | --- | --- |
| LI001 | 349 | 280 | 331 |  |
| LI010 | 0 | 1 | 1 |  |
| LI011 | 148 | 162 | 172 |  |
| LI012 | 0 | 0 | 0 |  |
| LI013 | 1 | 0 | 0 |  |
| LI018 | 1 | 1 | 1 |  |
| LI019 | 0 | 0 | 40 |  |
| LI020 | 1256 | 1816 | 2031 |  |
| LI021 | 0 | 0 | 1 |  |
| LI022 | 15 | 19 | 19 |  |
| LI023 | 108 | 63 | 65 |  |
| LI024 | 0 | 1 | 1 |  |
| LI025 | 3 | 1 | 1 |  |
| LI026 | 101 | 67 | 106 |  |
| LI027 | 53 | 39 | 36 |  |
| LI028 | 0 | 1 | 0 |  |
| LI029 | 2 | 3 | 4 |  |
| LI030 | 226 | 176 | 194 |  |
| LI031 | 0 | 0 | 1 |  |
| LI032 | 0 | 1 | 0 |  |
| LI033 | 1 | 0 | 1 |  |
| LI034 | 0 | 0 | 1 |  |
| LI035 | 0 | 1 | 0 |  |
| LI036 | 0 | 0 | 0 |  |
| LI037 | 0 | 0 | 1 |  |
| LI038 | 1 | 1 | 0 |  |
| LI039 | 1 | 3 | 5 |  |
| LI040 | 0 | 47 | 45 |  |
| LI041 | 1 | 2 | 2 |  |
| LI042 | 14 | 15 | 17 |  |
| LI045 | 2 | 2 | 2 |  |
| LI046 | 1 | 1 | 1 |  |
| LI047 | 1 | 0 | 0 |  |
| LI048 | 3139 | 3693 | 3625 |  |
| LI049 | 64 | 62 | 69 |  |
| LI050 | 0 | 1 | 1 |  |
| LI051 | 1 | 1 | 2 |  |
| LI052 | 0 | 0 | 0 |  |
| LI053 | 2 | 1 | 1 |  |
| LI054 | 2 | 6 | 7 |  |
| LI055 | 0 | 0 | 0 |  |
| LI056 | 30 | 28 | 33 |  |
| LI057 | 0 | 0 | 0 |  |
| LI058 | 1 | 1 | 0 |  |
| LI059 | 1 | 1 | 1 |  |
| LI060 | 1 | 1 | 2 |  |
| LI061 | 4 | 6 | 6 |  |
| LI062 | 2 | 1 | 2 |  |
| LI063 | 31 | 19 | 31 |  |
| LI064 | 2201 | 1361 | 1464 |  |
| LI066 | 2648 | 3046 | 3336 |  |
| LI071 | 243 | 233 | 263 |  |
| LI072 | 13 | 24 | 27 |  |
| LI073 | 86 | 271 | 373 |  |
| LI074 | 164 | 174 | 192 |  |
| LI075 | 1 | 1 | 2 |  |
| LI076 | 1 | 1 | 1 |  |
| LI077 | 8 | 9 | 10 |  |
| LI078 | 75 | 61 | 74 |  |
| LI079 | 13 | 12 | 12 |  |
