# Supplementary material for: Evaluation of cell count and classification capabilities in body fluids using a fully automated Sysmex XN equipped with high-sensitive Analysis (hsA) mode and DI-60 hematology analyzer system
Source: PLoS One. 2018 Apr 26;13(4):e0195923. doi: 10.1371/journal.pone.0195923 (PMC5919509; doi:10.1371/journal.pone.0195923)
Supplement: S2 Table — (DOCX) [file pone.0195923.s002.docx]

**S2 Table. Raw data for Fig 4A and B**

**(A)**

|  | NEUT% | | LYMP% | | EO% | | MONO% | |
| --- | --- | --- | --- | --- | --- | --- | --- | --- |
| Sample # | manual | DI-60 | manual | DI-60 | manual | DI-60 | manual | DI-60 |
| LI001 | 74.0 | 75.0 | 13.0 | 5.7 | 0.0 | 0.0 | 13.0 | 19.3 |
| LI011 | 68.5 | 70.1 | 22.0 | 11.8 | 2.0 | 1.6 | 7.5 | 16.6 |
| LI018 | 0.0 | 0.0 | 85.7 | 100.0 | 0.0 | 0.0 | 14.3 | 0.0 |
| LI020 | 84.5 | 91.2 | 13.0 | 5.0 | 0.0 | 0.0 | 2.5 | 3.8 |
| LI021 | 100.0 | 40.0 | 0.0 | 60.0 | 0.0 | 0.0 | 0.0 | 0.0 |
| LI022 | 2.0 | 0.6 | 83.0 | 71.2 | 0.0 | 0.6 | 15.0 | 27.6 |
| LI023 | 66.5 | 55.3 | 12.0 | 10.6 | 0.0 | 0.6 | 21.5 | 33.5 |
| LI024 | 33.3 | 25.0 | 41.7 | 58.3 | 8.3 | 4.2 | 16.7 | 12.5 |
| LI025 | 0.0 | 0.0 | 90.0 | 83.3 | 0.0 | 0.0 | 10.0 | 16.7 |
| LI026 | 9.5 | 11.7 | 63.5 | 67.9 | 0.0 | 0.0 | 27.0 | 20.4 |
| LI027 | 79.5 | 0.0 | 13.0 | 0.0 | 0.0 | 0.0 | 7.5 | 0.0 |
| LI028 | 0.0 | 0.0 | 100.0 | 100.0 | 0.0 | 0.0 | 0.0 | 0.0 |
| LI029 | 0.0 | 0.0 | 100.0 | 0.0 | 0.0 | 0.0 | 0.0 | 0.0 |
| LI030 | 92.0 | 0.0 | 5.0 | 0.0 | 0.0 | 0.0 | 3.0 | 0.0 |
| LI033 | 0.0 | 12.5 | 62.5 | 50.0 | 0.0 | 0.0 | 37.5 | 37.5 |
| LI038 | 14.8 | 0.0 | 73.9 | 74.2 | 0.0 | 0.0 | 11.3 | 25.8 |
| LI041 | 11.8 | 5.3 | 62.7 | 56.1 | 0.0 | 0.0 | 25.5 | 38.6 |
| LI042 | 3.0 | 0.0 | 86.0 | 87.3 | 1.0 | 1.2 | 10.0 | 8.7 |
| LI045 | 0.0 | 0.0 | 47.4 | 48.6 | 0.0 | 0.0 | 52.6 | 51.4 |
| LI048 | 88.5 | 92.3 | 8.5 | 6.3 | 2.0 | 1.4 | 1.0 | 0.0 |
| LI049 | 25.5 | 24.5 | 48.5 | 57.9 | 0.0 | 0.0 | 26.0 | 17.6 |
| LI055 | 0.0 | 0.0 | 100.0 | 100.0 | 0.0 | 0.0 | 0.0 | 0.0 |
| LI056 | 46.5 | 55.9 | 38.0 | 20.2 | 1.0 | 1.6 | 14.5 | 22.3 |
| LI059 | 0.0 | 0.0 | 75.0 | 75.0 | 0.0 | 0.0 | 25.0 | 25.0 |
| LI060 | 0.0 | 0.0 | 100.0 | 100.0 | 0.0 | 0.0 | 0.0 | 0.0 |
| LI061 | 0.0 | 0.0 | 76.0 | 74.3 | 0.5 | 0.0 | 23.5 | 25.7 |
| LI062 | 0.0 | 0.0 | 75.0 | 100.0 | 0.0 | 0.0 | 25.0 | 0.0 |
| LI064 | 73.5 | 67.2 | 1.0 | 3.6 | 0.0 | 0.0 | 25.5 | 29.2 |
| LI065 | 73.5 | 72.4 | 1.0 | 1.0 | 0.0 | 0.0 | 25.5 | 26.6 |
| LI066 | 77.0 | 87.2 | 5.0 | 2.7 | 0.0 | 0.0 | 18.0 | 10.1 |
| LI071 | 97.0 | 85.4 | 2.5 | 1.6 | 0.0 | 0.0 | 0.5 | 13.0 |
| LI072 | 7.0 | 5.5 | 86.0 | 67.2 | 7.0 | 8.6 | 0.0 | 18.8 |
| LI073 | 33.3 | 0.0 | 66.7 | 75.0 | 0.0 | 0.0 | 0.0 | 25.0 |
| LI074 | 54.5 | 37.3 | 24.0 | 38.9 | 0.0 | 0.6 | 21.5 | 23.1 |
| LI077 | 5.7 | 0.0 | 73.6 | 48.4 | 17.0 | 15.6 | 3.8 | 35.9 |
| LI078 | 4.5 | 6.8 | 61.0 | 19.3 | 26.5 | 35.4 | 7.0 | 37.9 |
| LI079 | 95.5 | 96.4 | 4.5 | 2.1 | 0.0 | 0.0 | 0.0 | 1.6 |

**(B)**

|  | NEUT% | | LYMP% | | EO% | | MONO% | |
| --- | --- | --- | --- | --- | --- | --- | --- | --- |
| Sample # | manual | DI-60 | manual | DI-60 | manual | DI-60 | manual | DI-60 |
| SP001 | 0.5 | 1.8 | 2.1 | 9.8 | 13.8 | 16.5 | 83.1 | 71.9 |
| SP002 | 7.5 | 9.1 | 19.6 | 17.8 | 30.7 | 27.4 | 41.7 | 45.7 |
| SP004 | 14.0 | 15.5 | 75.0 | 66.5 | 0.5 | 0.0 | 10.5 | 17.5 |
| SP005 | 9.5 | 16.9 | 69.0 | 65.7 | 0.5 | 0.0 | 21.0 | 17.4 |
| SP007 | 9.5 | 11.0 | 78.4 | 85.7 | 2.5 | 0.0 | 9.5 | 3.3 |
| SP008 | 1.0 | 0.6 | 11.1 | 9.8 | 0.0 | 0.0 | 87.9 | 89.6 |
| SP009 | 0.0 | 0.5 | 9.0 | 16.9 | 0.0 | 0.0 | 91.0 | 82.5 |
| SP010 | 0.5 | 0.0 | 74.7 | 73.3 | 0.0 | 0.0 | 24.7 | 26.7 |
| SP011 | 5.3 | 14.3 | 62.1 | 78.6 | 2.1 | 0.0 | 30.5 | 7.1 |
| SP013 | 2.0 | 1.0 | 20.0 | 16.1 | 68.0 | 73.4 | 10.0 | 9.5 |
| SP014 | 2.5 | 0.0 | 18.6 | 13.0 | 0.0 | 0.0 | 78.9 | 87.0 |
| SP015 | 0.7 | 0.0 | 51.7 | 61.6 | 2.6 | 0.0 | 45.0 | 38.4 |
| SP016 | 5.1 | 3.3 | 54.3 | 83.0 | 0.6 | 0.0 | 40.0 | 13.7 |
| SP020 | 6.9 | 2.1 | 27.7 | 30.3 | 0.0 | 0.0 | 65.4 | 60.6 |
| SP022 | 1.7 | 3.8 | 12.1 | 14.6 | 0.0 | 0.0 | 86.2 | 81.6 |
| SP027 | 15.7 | 25.4 | 46.2 | 32.0 | 8.6 | 8.8 | 29.4 | 33.7 |
| SP034 | 0.0 | 0.0 | 91.5 | 42.8 | 0.0 | 0.0 | 8.5 | 57.2 |
| SP036 | 12.0 | 0.0 | 72.0 | 57.4 | 4.0 | 1.9 | 12.0 | 40.7 |
| SP037 | 0.0 | 0.0 | 0.0 | 22.6 | 0.0 | 1.6 | 100.0 | 75.8 |
| SP039 | 3.0 | 9.3 | 42.6 | 46.5 | 0.0 | 0.0 | 54.3 | 44.2 |
| SP042 | 1.5 | 0.0 | 36.4 | 13.3 | 0.0 | 0.0 | 62.1 | 86.7 |
| SP044 | 17.6 | 12.3 | 60.8 | 63.6 | 18.1 | 21.4 | 3.5 | 0.5 |
| SP045 | 11.0 | 12.8 | 56.5 | 54.0 | 30.5 | 23.0 | 2.0 | 10.2 |
| SP046 | 1.0 | 0.0 | 55.6 | 40.6 | 0.0 | 0.0 | 43.4 | 59.4 |
| SP047 | 1.0 | 0.0 | 41.5 | 43.5 | 0.0 | 0.0 | 0.0 | 56.5 |
| SP049 | 23.5 | 22.6 | 36.1 | 18.2 | 0.5 | 0.0 | 39.9 | 59.1 |
| SP050 | 31.3 | 29.9 | 20.7 | 20.1 | 0.0 | 0.0 | 48.0 | 50.0 |
| SP052 | 90.5 | 78.1 | 6.0 | 7.5 | 0.0 | 0.0 | 3.5 | 14.4 |
| SP056 | 5.5 | 0.4 | 40.2 | 77.2 | 6.0 | 0.0 | 48.2 | 22.3 |
| SP057 | 3.0 | 1.4 | 48.7 | 49.8 | 3.0 | 4.1 | 45.2 | 44.3 |
| SP059 | 8.0 | 19.7 | 56.0 | 56.7 | 0.5 | 0.0 | 35.5 | 23.6 |
| SP060 | 15.6 | 13.0 | 19.6 | 20.9 | 1.0 | 0.0 | 63.3 | 66.1 |
| SP063 | 55.9 | 29.6 | 2.8 | 33.8 | 0.0 | 9.2 | 41.2 | 27.5 |
| SP064 | 70.6 | 65.5 | 16.5 | 13.2 | 1.0 | 0.0 | 11.9 | 21.3 |
| SP067 | 0.0 | 1.5 | 72.3 | 60.7 | 0.0 | 0.0 | 27.7 | 37.8 |
| SP068 | 2.6 | 2.1 | 89.1 | 85.0 | 0.5 | 2.1 | 6.8 | 8.8 |
| SP076 | 0.5 | 1.3 | 88.6 | 82.6 | 0.0 | 0.0 | 10.9 | 16.1 |
| SP077 | 1.5 | 1.6 | 95.5 | 87.5 | 0.0 | 0.0 | 3.0 | 10.9 |
| SP079 | 51.0 | 17.7 | 7.5 | 0.0 | 10.5 | 1.0 | 31.0 | 81.3 |
| SP081 | 13.7 | 4.3 | 55.3 | 84.4 | 0.0 | 0.0 | 31.0 | 11.3 |
| SP082 | 6.2 | 1.9 | 73.0 | 65.6 | 0.0 | 0.0 | 20.8 | 32.5 |
| SP083 | 1.0 | 0.0 | 98.0 | 97.9 | 0.0 | 0.0 | 1.0 | 2.1 |
| SP084 | 1.5 | 0.0 | 24.6 | 43.2 | 0.0 | 0.0 | 73.8 | 56.8 |
| SP085 | 24.0 | 15.9 | 39.5 | 56.6 | 2.5 | 3.5 | 33.5 | 23.9 |
| SP086 | 33.0 | 7.6 | 55.0 | 89.4 | 3.5 | 0.0 | 8.5 | 3.0 |
| SP087 | 9.6 | 3.6 | 3.0 | 4.4 | 0.0 | 0.0 | 12.6 | 91.9 |
| SP088 | 17.1 | 11.7 | 41.7 | 71.2 | 1.5 | 2.3 | 39.7 | 14.9 |
| SP089 | 2.0 | 0.0 | 40.7 | 20.5 | 0.5 | 0.0 | 56.8 | 79.5 |
| SP090 | 4.3 | 5.6 | 76.0 | 83.1 | 0.0 | 0.0 | 92.4 | 11.3 |
| SP091 | 47.2 | 44.1 | 19.3 | 16.9 | 11.2 | 4.6 | 21.8 | 34.4 |
| SP092 | 0.9 | 5.8 | 34.0 | 30.1 | 2.8 | 5.2 | 62.3 | 59.0 |
| SP093 | 6.0 | 26.1 | 84.5 | 55.9 | 0.0 | 0.5 | 9.5 | 17.6 |
| SP094 | 1.5 | 0.0 | 67.8 | 20.0 | 0.0 | 0.0 | 30.7 | 80.0 |
| SP095 | 4.8 | 1.0 | 32.1 | 47.3 | 0.0 | 1.0 | 63.1 | 50.7 |
| SP096 | 0.0 | 0.0 | 99.5 | 98.4 | 0.5 | 0.0 | 0.0 | 1.6 |
| SP097 | 38.6 | 38.0 | 21.3 | 18.5 | 8.1 | 7.0 | 32.0 | 36.5 |
| SP098 | 7.5 | 10.5 | 43.7 | 37.6 | 0.5 | 0.5 | 48.2 | 51.0 |
| SP099 | 29.5 | 16.8 | 14.5 | 21.1 | 0.0 | 0.0 | 56.0 | 62.1 |
| SP103 | 28.1 | 18.8 | 8.5 | 26.7 | 31.7 | 4.0 | 31.7 | 50.5 |
| SP104 | 0.0 | 0.5 | 11.0 | 79.9 | 3.0 | 0.0 | 10.5 | 19.6 |
